# Health-Related Quality of Life and Depressive Symptomatology in High School Students during the Lockdown Period Due to SARS-CoV-2

**DOI:** 10.3390/ijerph19148780

**Published:** 2022-07-19

**Authors:** Guillermo Gómez-Delgado, Ernesto Almaraz-Vega, Jaime Eduardo Ramírez-Mireles, María Elena Gutiérrez-Paredes, María del Rocío Padilla-Galindo

**Affiliations:** 1 Natural and Health Sciences, Tepatitlan Regional Preparatory School, High School Education System, University of Guadalajara, Guadalajara 47600, Mexico; ernesto.almaraz5309@academicos.udg.mx (E.A.-V.); jaime.ramirez@sems.udg.mx (J.E.R.-M.); maria.gutierrez2361@academicos.udg.mx (M.E.G.-P.); 2 University Center of the South, University of Guadalajara, Guadalajara 49000, Mexico; maria.galindo@cusur.udg.mx

**Keywords:** SARS-CoV-2, lockdown period, depressive symptomatology, health-related quality of life, high school students

## Abstract

The objectives of this study were to evaluate and compare the prevalence of health-related quality of life and depressive symptomatology in high school students during the lockdown period due to the SARS-CoV-2 pandemic. A cross-sectional survey was conducted with students attending the High School Education System of the University of Guadalajara, Jalisco, México. Through a Google Forms survey, students answered their perceptions of health-related quality of life and depressive symptomatology. The outcome variable was the presence of depressive symptoms, assessed using the Children’s Depression Inventory (CDI). Cronbach’s alpha coefficient was 0.8 in both surveys. A total of 1446 students participated (women, 64.9%; mean age of 16.1 ± 0.9 years). Among the students, 22% manifested clinical depressive symptoms (24.4 ± 5.0), and males showed lower scores on health-related quality of life and depressive symptoms (44.9 ± 11.9, *p* = 0.005) (12 ± 7.7, *p* = <0.001) compared to their female peers (45.2 ± 10.6, *p* = 0.005) (13.7 ± 7.5, *p* = <0.001), respectively. During the lockdown due to the SARS-CoV-2 pandemic, a high prevalence of depressive symptomatology was identified in our students with in addition to a low perception of health-related quality of life in dimensions, mood and emotions, and peers and social support.

## 1. Introduction

In late December 2019, in the city of Wuhan, Hubei Province, China, a series of cases were reported that met the criteria for pneumonia of unknown etiology and severe characteristics [1]; it was identified and reported as a novel coronavirus disease in bronchoalveolar-lavage fluid samples from three patients, named 2019-nCoV [2]. Due to its multidimensional impact and its high risk of spreading to other countries, it was considered an international public health emergency and by March 2020, it reached the level of a pandemic [3].

On the 27th of February 2020, the first confirmed positive case of severe acute respiratory syndrome coronavirus 2 (SARS-CoV-2) was confirmed in Mexico [4]; in the state of Jalisco, due to the increase in contagions, lockdown and social distancing was issued as a mandatory health security measure to prevent and contain the spread and transmission of SARS-CoV-2. The University of Guadalajara implemented the suspension of classroom teaching to reduce the risks of infection among staff and students on the 17th of March 2020, and in the students, the measure implied the loss of their natural play and learning environment.

In times of pandemic and environmental disasters, which increase the risk of post-traumatic stress, depression, and anxiety [5], children and adolescents may be highly exposed to biopsychosocial stressors generated by the disruption in their daily life routine as a result of social isolation and their unseasoned ability to conceive and comprehend the short-and long-term consequences of the outbreak [6].

During the SARS-CoV-2 outbreak, a high prevalence of psychological health problems (depressive and anxiety symptoms) has been described, which among adolescents was negatively associated with the level of awareness of SARS-CoV-2 [2,7]. Other studies have shown the impact on mental health in adolescents during the SARS-CoV-2 pandemic such as psychological problems and post-traumatic stress [8] and depressive symptomatology [9], and have shown how the implementation of restrictions that caused social isolation, loss of routine, and how widespread misinformation in the media amplified perceptions of the risk of a mental health crisis [10]. On the other hand, it was found that mental health problems in students were attenuated and were less frequent among those who felt virtually connected to their peers [11].

In adolescents, lockdown measures to control and prevent the SARS-CoV-2 pandemic can negatively impact on their health-related quality of life (HRQoL) which is a subjective construct that evaluates the perceived health of an individual on the sub-dimensions of life, including physical, psychological, social functioning, and wellbeing [12]. A quantitative study evaluated the HRQoL of the Norwegian adolescents during the SARS-CoV-2, and the results showed a low HRQOL compared to European parameters and concluded that being in quarantine, having been confirmed or suspected of SARS-CoV-2, were negatively associated with the HRQoL [13]. Other evidence evaluated the impact of health literacy on depression and the HRQoL, identifying that people with SARS-CoV-2 had a higher probability of depression and low HRQoL scores [14,15], and a systematic review showed that SARS-CoV-2 could have significantly decreased the HRQoL of children and adolescents [16].

On the other hand, we can currently find that many countries have decided to return to face-to-face classes in order to minimize the problems generated by the confinement and, in addition to the fact that there is now a vaccine for SARS-CoV-2, it is intended that normality will slowly return, which raises new questions related to the return to face-to-face classes, but at the same time, new studies with relevant information has been generated in recent months [17,18]; for example, Puteikis and collaborators found that going back to school has a direct negative impact on the quality of sleep of students, but at the same time, it is beneficial in the academic development and physical activity of students [19].

There are theoretical reasons and scientific findings which suggest that depressive symptomatology and HRQoL could be involved in the cascade of psychoemotional disturbances that adolescents face during the pandemic, and the present study focused on students enrolled in the Tepatitlan Regional Preparatory School (TRPS) of the University of Guadalajara, with the objective of evaluating and comparing the prevalence of the HRQoL profile and depressive symptomatology manifestations during the lockdown and social distancing by the SARS-CoV-2 pandemic.

## 2. Materials and Methods

### 2.1. Study Type and Participants

Through a cross-sectional descriptive exploratory study, during the months of May and June 2020, students enrolled at the TRPS and its campuses (Acatic, Cañadas de Obregon, Valle de Guadalupe, and Yahualica, Jalisco, Mexico), were selected by a non-probabilistic sampling of available subjects. Students who did not present the signed letter of consent and from the sixth semester were excluded, considered as a possible bias related to the level of stress and anxiety they experience upon graduating from high school.

### 2.2. Context and Data Collection Procedure

The TRPS is an urban public high school at the University of Guadalajara, it has an academic program based on a general baccalaureate on competences; students from 5 campuses of the TRPS located in different urban cities named Acatic, Cañadas de Obregon, Valle de Guadalupe, and Yahualica, in Jalisco, Mexico, were invited to participate.

### 2.3. Data Collection Procedure

Due to health contingency, for data collection, the psychometrics were converted into Google Forms, which were disseminated by the group’s tutor on various virtual platforms, social networks, and the student’s email. Those interested in participating were informed about the nature and objective of the research, the principle of anonymity, and were notified that they could decline at the time they considered it pertinent without affecting their academic grades. The students who freely and voluntarily decided to participate digitally sent the informed consent forms signed by the parent or guardian and thus proceeded to enter the access links to the psychometric tests.

### 2.4. Instruments

The HRQoL profile was evaluated through the KIDSCREEN-52 questionnaire, and translated and cross-culturally adapted for the Mexican population by Hidalgo, Rajmil, and Montaño [20]; the 52-item version measures 10 dimensions of HRQoL: physical well-being, psychological well-being, mood and emotions, self-perception, autonomy, parent relations and home life, financial resources, social support and peers, school environment, and social acceptance [20]. The answer options were categorized on a 5-option Likert scale, which identifies the frequency or intensity of the attribute during the period of the week prior to the application [21], and the mean scores of each dimension were calculated and standardized to a mean of 50 and a standard deviation (SD) of 10; for their interpretation, the score obtained by an individual (range: 1–5) in the dimension was considered, as well as the mean and standard deviation of the reference group. Subsequently, the dimensions were transformed into dichotomous variables taking the score corresponding to 0.8 SD below the mean of 42 points as a cut-off point; scores below 42 constituted the category of worst HRQoL in the corresponding dimension [20,22].

To determine the presence or severity of depressive symptomatology, the Children Depression Inventory (CDI) [23] was applied, a 27-item questionnaire that evaluates two scales: dysphoria (depressive mood, sadness, worry, etc.) and negative self-esteem (judgments of ineffectiveness, ugliness, badness, etc.). Each item consists of three alternative answers with values of 0, 1, and 2, where the higher score indicates a greater degree of risk of depressive symptomatology. In general, the total score has a range between 0 and 54 points, when the total CDI score of ≥19 is related to depressive clinical symptomatology; scores of 12–18 suggest subclinical depression; and lower than 12 are considered normal [24].

### 2.5. Data Analysis

The statistical analysis was performed in the computer package Sigma Plot Statistics version 14.0, descriptive statistics were obtained, and the psychometric properties of reliability were analyzed (Cronbach’s alpha ≥ 0.7 considered satisfactory). A Pearson correlation was applied for the different variables, Student’s *t*-test was used to determine significant differences between the means of two groups and multivariate linear regression models took the scores of each of the KIDSCREEN-52 dimensions as independent variables and the scores of the CDI domains as dependent variables; in all analyses, a statistical significance level of less than 0.05 was taken.

## 3. Results

In the months of May–June 2020, 1446 students (35.16% of the school enrollment) answered, completed, and submitted the psychometric questionnaires. The average age of 16.1 ± 0.8 was identified (Table 1); concerning sex, 64.9% of females were evaluated with an average age of 16.1 ± 0.9 and 35.1% of males with 16.4 ± 0.8. 64.1% of students from TRPS Campus, 11.3% from Acatic Campus, 5.4% from Cañadas de Obregon Campus; 10.4% from Valle de Guadalupe Campus, and 8.8% from Yahualica Campus participated.

The evaluation of the reliability of both scales was excellent, with Cronbach’s alpha scores greater than 0.8, with the exception of the self-perception and social acceptance dimensions (Cronbach’s alpha = 0.77) (Table 2); the mean scores of the CDI and the KIDSCREEN-52 corroborated the expected differences with respect to gender; in males, statistically, significantly lower scores were observed compared to their female peers in the CDI scale and in three of the dimensions of the KIDSCREEN-52 (Table 2).

With respect to the manifestations of depressive symptomatology, it was identified that 22.1% (319 students) of the students presented clinical depressive symptomatology (24.4 ± 5.0), and 29.3% presented subclinical depressive symptomatology (14.7 ± 2.0) and 48.6% of the students did not present depressive symptomatology (6.8 ± 2.7).

Table 1 shows the results obtained from the psychometric characteristics corresponding to the different campuses where the test was applied, it is observed that the average age between the different campuses is very similar, and the difference is in the sample size: 1446 students took the test in Tepatitlan Regional Preparatory School Campus while only 77 took the test in the Cañadas de Obregon Campus. It is important to mention that the Tepatitlan Regional Preparatory School Campus is the campus with the largest number of students, which explains the disproportionality shown in the table.

The HRQoL profile of students was good, and the highest scores corresponded to the dimensions, physical well-being, psychological well-being, self-perception, and parent relation and home life in contrast to the dimension’s autonomy, mood and emotions, and social support and peers in which the central values identified were 43.3 ± 10.1, 41.9 ± 12.1, and 41.4 ± 11.8, respectively (Table 1).

A significant statistical relationship was observed in most of the dimensions of the KIDSCREEN-52-CDI (*p* < 0.01), with a negative association evidencing a worse HRQoL in students with higher scores in the dimensions of depressive symptomatology, except for the dimensions of financial resources and social support and peers (*p* = 0.07 and 0.19, respectively); moreover, a low negative correlation was identified between the KIDSCREEN-52-CDI variables (Table 3).

When we were running the multivariate adjustment, statistical significance was found between the variables associated with the mood and emotions dimension, school environment, and social acceptance—allowing us to identify them as potential predictive factors in the study population (Table 4). It is important to mention that these dimensions are closely related to the social interactions that students experiment in the classroom; thus, when this interaction is almost completely eliminated, the self-esteem is affected, and a feeling of dysphoria is generated in the student.

Table 5 shows that the ANOVA obtained for the regression, it can be observed that although an R-squared of 0.028 was obtained (Table 6), the model is statistically significant, it is important to point out that the model can be improved, but it was decided to keep all the variables of the Kidscreen-52 test in order to compare in the first instance the effect of each one of them with respect to the CDI.

## 4. Discussion

As the new disease caused by SARS-CoV-2 rapidly spread around the world, public health experts suggested that university and government authorities implement preventive measures, including social distancing, isolation, or quarantine in order to reduce the spread of the virus; an ambivalent decision that entailed an effective benefit and adverse effects by drastically disrupting society’s way of life.

During the time of the pandemic, adolescents are especially vulnerable to states of anxiety and depressive symptoms [25]; the decree of social distancing implied the closure of schools and the impossibility for schoolchildren to interact with their peers [26]; for our students, staying at home was related to fear of contagion, frustration, irregular sleep patterns, prolonged exposure to mobile devices, sedentary lifestyle, and catastrophic thoughts due to bad information. In addition to the above, students had to migrate their classes to virtual environments (online classes), all of which were problems that together had an impact on stress and anxiety indicators, thus adding to the psycho-emotional complications of adolescents and the intrinsic and extrinsic factors inherent in the SARS-CoV-2 pandemic, ultimately having a negative impact on the mental health of adolescents.

The results of our research reveal an alarming prevalence of clinical symptoms of depression (22.1%) similar to what was reported in previous research [9,27]; a high prevalence of depressive symptomatology was identified in the studied population, a finding that exhibits the manifestations of students’ mental health when they are confronted to contexts of vulnerability. It was observed that the prevalence of depressive symptomatology in the studied population was high compared to recent studies in the absence of epidemics [28,29]; however, it is important to compare the effect that confinement had during the beginning of the pandemic in our institution, so it is intended in the near future to perform the test again in a post-pandemic period.

The KIDSCREEN-52 test presented good reproducibility and validity properties with Cronbach’s alpha result greater than 0.7 in all its dimensions, in agreement with previous reports [20,22,30]. In general, an average of 44.9 was identified in the ten dimensions that evaluate psychometrics, which translates into an adequate perception of the HRQoL of our students under conditions of vulnerability and which coincides with previous research [24,31,32]; results that could be associated with the cultural perception of the quality of life in health, to the same definition of the concept of health and even to the impact of the pandemic on the environment that adolescents interact with [32].

Particularly, the mood and emotions dimension refers to a low level of CVRS (41.9 ± 12.1), as well as the social support and peers dimension (41.4 ± 11.8); findings that could be correlated to the peculiar circumstance that caused the sudden interruption of daily life and the mandatory home confinement typical of quarantine and its collateral effects, which is consistent with previous reports [14,15].

Regarding the dimensions evaluated by KIDSCREEN-52 and analyzing the fact that scores equal to or lower than 42 constitute categories of worse CVRS, it was observed that 31.8% of the population presented a low perception of quality of life in the dimension of physical well-being, a finding correlated with the lack of physical activity. Among the students, 33.3% manifested a low perception of CVRS in the dimension of psychological well-being. In the mood and emotions dimension, 49.3% of our students manifested negative experiences, depressive states, and stressful emotions during a period of social distancing.

In the self-perception dimension, 33.9% of schoolchildren reported dissatisfaction related to body image; in the dimension of autonomy, 43% reported restrictions in recreational activities and participation in social activities; and in the dimension of relationship with parents and family life, 33.8% of schoolchildren reported feeling lonely, ignored at home and not appreciated by their parents or guardians. This could be associated with the fact that many of the parents or tutors decreased their income, which in some cases lost their jobs and most of them felt the fear related to panic buying and limited access to food in department stores.

It was observed that, in the dimension of social support and peers, 39.1% (*n* = 566) (35.6 ± 5.6) of our students reported a low quality of life profile for the respective dimension, which implies the fact of exclusion and non-acceptance by their peers. In the school environment dimension, 49.6% (*n* = 718) (31.7 ± 7.2) of our students referred school dissatisfaction, which could be related to the school transition experienced by students when radically changing from face-to-face learning to online learning with the support of social networks or virtual classes.

Among the students, 41.6% (*n* = 602) (34.2 ± 6.1) reported feeling rejected by their peers and 36.9% (*n* = 534) (30.1 ± 9.6) reported a low quality of life in the financial resources dimension, a fact that is correlated with the adverse consequences of confinement due to the pandemic.

We consider among the limitations of this study its inability to generalize the results in the high school population; in our institution, there have not been previous studies related to the profile of the CVRS and depressive symptomatology of its students, and therefore it is not possible to establish pre- and post-pandemic comparisons, another limitation related to the fact that the present work represented a cross-sectional study so it is not possible to establish cause–effect relationships in the studied variables and due to the impossibility of anticipating the evolution of the pandemic. This is as a limitation which prevent the planning and a second application of psychometrics in a non-pandemic environment.

## 5. Conclusions

The results of this study confirm the suspicions that the pandemic caused by SARS-CoV-2 and the lockdown measures have negative effects on the mental health of adolescents: students reported through psychometrics a high prevalence of clinical and subclinical depressive symptomatology; in general, an adequate perception of the health-related quality of life in conditions of vulnerability was identified, except in mood and emotion, and social support and peers’ dimensions, in which students reported a low perception of health-related quality of life.

Moreover, these results suggest that appropriate mental healthcare delivery should be at the center of future programs of TRPS, which allow us to anticipate, plan, and execute strategies for the protection of mental health in the student community.

Our findings highlight an area of opportunity for the education system managers of the University of Guadalajara who could develop and promote programs and strategies for the promotion and the care of mental health throughout the whole university community to reduce the psychological damage caused by social distancing during the pandemic.

## Figures and Tables

**Table 1:** Psychometric characteristics of the sample (*n* = 1446), stratified by campus.

Variables	TOTAL Mean ± SD (Min–Max)	TRPS Campus * Mean ± SD (Min–Max)	A Campus * Mean ± SD (Min–Max)	CO Campus * Mean ± SD (Min–Max)	VG Campus * Mean ± SD (Min–Max)	Y Campus * Mean ± SD (Min–Max)
*n*	1446	927	164	77	151	127
Age	16.1 ± 0.8 (15–20)	16.1 ± 0.9 (15–19)	16.4 ± 0.8 (15–20)	15.9 ± 0.7 (15–18)	16 ± 0.7 (15–20)	15.7 ± 0.6 (15–17)
Children Depressive Inventory						
Total Dysphoria Negative self-esteem	13.1 ± 7.6 (1–45)	13.4 ± 8.1 (1–45)	13.3 ± 6.1 (2–33)	8.9 ± 6.2 (1–31)	13.6 ± 7.2 (3–43)	12.5 ± 6.1 (1–27)
	8.37 ± 5.0 (0–28)	8.58 ± 5.3 (0–28)	8.29 ± 4.2 (2–23)	5.84 ± 4.2 (0–20)	8.41 ± 4.6 (0–25)	8.36 ± 4.1 (1–19)
	4.74 ± 3.3 (0–18)	4.83 ± 3.5 (0–18)	4.91 ± 2.5 (0–11)	3.05 ± 2.4 (0–12)	5.23 ± 3.1 (0–18)	4.31 ± 2.7 (0–11)
KIDSCREEN-52						
Physical well-being (PHWB)	48.1 ± 11.3 (15.6–67.3)	48.1 ± 11.3 (15.6–67.3)	47.2 ± 10.4 (18.5–67.3)	53.3 ± 12.8 (18.5–67.3)	47.1 ± 10.9 (24.2–67.3)	48.1 ± 11.4 (18.5–67.3)
Psychological well-being (PWB)	47 ± 10.9 (10.7–63.8)	46.7 ± 11.4 (10.7–63.8)	47.2 ± 9.3 (21.8–63.8)	51.3 ± 10.8 (21.8–63.8)	46.4 ± 10 (14.4–63.8)	47 ± 9.2 (24–63.8)
Mood and emotions (ME)	41.9 ± 12.1 (6.7–63.1)	41.3 ± 12.7 (6.7.63.1)	42.6 ± 10 (14.8–63.1)	43.3 ± 11.2 (24.8–63.1)	41.4 ± 11.3 (6.7–61.1)	41.9 ± 9.9 (18.8–63.1)
Self-perception (SP)	46.8 ± 9.9 (17.9–64.3)	46.6 ± 10.1 (17.9–64.3)	46.2 ± 9.7 (27.2–64.3)	50.9 ± 9.4 (27.2–64.3)	45 ± 9.5 (22.6–64.6)	48.1 ± 9.1 (22.6–64.3)
Autonomy (A)	43.3 ± 10.1 (17–63.5)	42.8 ± 10.3 (17–63.5)	43.1 ± 8.8 (21.6–63.5)	48.7 ± 9.8 (23.9–63.5)	43.7 ± 9.8 (21.6–63.5)	43.2 ± 9.7 (19.3–63.5)
Parent relation and home life (PRHL)	46.7 ± 11.8 (12.2–61.6)	46.2 ± 12.3 (12.2–61.6)	45.7 ± 11.2 (18.4–61.6)	50.8 ± 9.8 (26.6–61.6)	47.7 ± 10.8 (14.3–61.6)	47.9 ± 10.6 (20.5–61.6)
Financial resources (FR)	45.4 ± 9.5 (22–60.6)	44.9 ± 9.7 (22–60.6)	43.5 ± 9 (25.5–60.6)	48.3 ± 9.1 (25.2–60.6)	46.9 ± 8.4 (22–60.6)	47.6 ± 9.5 (22–60.6)
Social support and peers (SSP)	41.4 ± 11.8 (12.4–63.7)	40.8 ± 12.1 (12.4–63.7)	40.2 ± 10.7 (12.4–63.7)	46 ± 11.3 (18.8–63.7)	42.9 ± 11 (14.5–63.7)	43 ± 10.8 (12.4–63.7)
School environment (SE)	44.7 ± 10.8 (19.4–68.4)	44.7 ± 11.1 (19.4–68.4)	44.7 ± 9 (21.4–68.4)	50.8 ± 9.3 (23.5–64.8)	41.3 ± 10.2 (19.4–66.3)	45.2 ± 0.6 (19.4–68.4)
Social acceptance (SA)	44.3 ± 12.3 (−7.6–56.3)	44.3 ± 12.5 (−7.6–56.3)	42.3 ± 13.6 (3–56.3)	46.5 ± 10.3 (8.4–56.3)	43 ± 11.7 (8.4–65.3)	46.8 ± 9.6 (13.7–56.3)

* Acronyms: SD—Standard deviation; Min–Max—Minimun–Maximun; TRPS Campus—Tepatitlan Regional Preparatory School Campus; A Campus—Acatic Campus; CO Campus—Cañadas de Obregon Campus; VG Campus—Valle de Guadalupe Campus; Y Campus—Yahualica Campus.

**Table 2:** Cronbach’s alpha coefficient (CDI-KIDSCREEN-52) stratified by gender.

Variables	TOTAL Mean ± SD (Cronbach’s Alpha Coefficient)	Females Mean ± SD	Males Mean ± SD	*t*-Student	*p*-Value
*n*	1446	938	508		
Age	16.1 ± 0.8	16.1 ± 0.9	16.4 ± 0.8		
Children Depressive Inventory					
Total Dysphoria Negative self-esteem	13.1 ± 7.6 (0.88)	13.7 ± 7.5	12 ± 7.7	3.505	*p* ≤ 0.001
	8.38 ± 5.0 (0.81)	8.80 ± 5.0	7.55 ± 4.9	4.521	*p* ≤ 0.001
	4.74 ± 3.3 (0.82)	4.84 ± 3.2	4.56 ± 3.4	1.476	*p* = 0.140
KIDSCREEN-52					
Physical well-being (PHWB)	48.1 ± 11.3 (0.80)	48 ± 11	49.7 ± 11.5	−2.656	*p* = 0.008
Psychological well-being (PWB)	47 ± 10.9 (0.90)	47.8 ± 10.2	46.6 ± 12.2	2.118	*p* = 0.034
Mood and emotions (ME)	41.9 ± 12.1 (0.90)	42.7 ± 11	41.5 ± 14.1	1.744	*p* = 0.081
Self-perception (SP)	46.8 ± 9.9 (0.77)	48.4 ± 9.6	45.3 ± 11.2	5.560	*p* ≤ 0.001
Autonomy (A)	43.3 ± 10.1 (0.81)	44.2 ± 9.6	42.4 ± 11.2	3.208	*p* = 0.001
Parent relation and home life (PRHL)	46.7 ± 11.8 (0.91)	47.3 ± 11.1	46.2 ± 13.1	1.698	*p* = 0.090
Financial resources (FR)	45.4 ± 9.5 (0.85)	44.9 ± 9.3	46.5 ± 9.7	−3.079	*p* = 0.002
Social support and peers (SSP)	41.4 ± 11.8 (0.85)	40.9 ± 11.4	42 ± 12.5	−1.673	*p* = 0.095
School environment (SE)	44.7 ± 10.8 (0.88)	44.8 ± 10.7	44.3 ± 11.2	0.862	*p* = 0.005
Social acceptance (SA)	44.3 ± 12.3 (0.77)	43.3 ± 12.6	45.3 ± 12.5	−2.832	*p* ≤ 0.005

Acronyms: SD—standard deviation.

**Table 3:** Pearson’s correlation coefficients between the HRQoL and CDI.

KIDSCREEN-52	CDI					
	General	*p*-Value	Dysphoria	*p*-Value	Negative Self-Esteem	*p*-Value
*PHWB*	−0.072	0.00	−0.063	0.01	−0.077	0.00
*PWB*	−0.108	0.00	−0.098	0.00	−0.107	0.00
*ME*	−0.137	0.00	−0.127	0.00	−0.129	0.00
*SP*	−0.109	0.00	−0.096	0.00	−0.112	0.00
*A*	−0.059	0.02	−0.063	0.01	−0.044	0.09
*PRHL*	−0.100	0.00	−0.088	0.00	−0.103	0.00
*FR*	−0.047	0.07	−0.050	0.05	−0.034	0.19
*SSP*	−0.034	0.19	−0.043	0.09	−0.018	0.49
*SE*	−0.119	0.00	−0.099	0.00	−0.131	0.00
*SA*	−0.112	0.00	−0.097	0.00	−0.115	0.00

Acronymous: CDI—Children Depressive Inventory; PHWB—physical well-being; PWB—psychological well-being; ME—mood and emotions; SP—self-perception; A—autonomy; PRHL—parent relation and home life; FR—financial resources; SSP—social support and peers; SE—school environment; SA—social acceptance.

**Table 4:** Regression coefficients for the dimensions of health-related to the quality of life.

Independent Variable	Dependent Variable CDI		
	Coefficients	95% CI	*p*-Value
*PHWB*	0.013	[−0.034, 0.061]	0.580
*PWB*	−0.005	[−0.070, 0.061]	0.891
*ME*	−0.060	[−0.118, −0.003]	0.038
*SP*	−0.033	[−0.087, 0.021]	0.235
*A*	0.019	[−0.035, 0.073]	0.489
*PRHL*	−0.004	[−0.054, 0.045]	0.864
*FR*	0.004	[−0.045, 0.053]	0.881
*SSP*	0.013	[−0.030, 0.056]	0.562
*SE*	−0.056	[−0.099, −0.013]	0.011
*SA*	−0.040	[−0.073, −0.007]	0.018

Acronymous: CDI—Children Depressive Inventory; PHWB—physical well-being; PWB—psychological well-being; ME—mood and emotions; SP—self-perception; A—autonomy; PRHL—parent relation and home life; FR—financial resources; SSP—social support and peers; SE—school environment; SA—social acceptance; CI—confidence intervals.

**Table 5:** ANOVA for regression analysis.

Source of Variation	df	SS	MS	F	Significance F
Regression	10	2558.55757	255.855757	4.23681771	7.924 × 10^−6^
Residual	1419	85,691.5131	60.3886632		
Total	1429	88,250.0706			

**Table 6:** Regression summary output.

Regression of Statistics	
Multiple R	0.170
R square	0.028
Adjusted R squared	0.022
Standard error	7.771
Observations	1430

## Data Availability

Due to sensitive data, the data can be accessed upon request to the authors.

## References

[1] Aragón R., Vargas I., Miranda M.G. (2020). COVID-19 by SARS-CoV-2: The new health emergency. Mex. J. Pediatr..

[2] Zhu N., Zhang D., Wang W., Li X., Yang B., Song J., Zhao X., Huang B., Shi W., Lu R. (2020). A novel coronavirus from patients with pneumonia in China, 2019. N. Engl. J. Med..

[3] Rajkumar R.P. (2020). COVID-19 and mental health: A review of the existing literature. Asian J. Psychiatry.

[4] Suárez V., Suarez M., Oros S., Ronquillo E. (2020). Epidemiology of COVID-19 in Mexico: From the 27th of February to the 30th of April 2020. Rev. Clínica Española.

[5] Douglas P.K., Douglas D.B., Harrigan D.C., Douglas K.M. (2009). Preparing for pandemic influenza and its aftermath: Mental health issues considered. Int. J. Emerg. Ment. Health.

[6] Saggioro C., Capucho P., Lima L.C., Mázala T., da Silva L., Roany I., Soares E., de Araujo E.G., Araujo A., Oliveira P. (2021). COVID-19 pandemic impact on children and adolescents’ mental health: Biological, environmental, and social factors. Prog. Neuro-Psychopharmacol. Biol. Psychiatry.

[7] Odriozola P., Planchuelo Á., Irurtia M.J., de Luis R. (2020). Psychological effects of the COVID-19 outbreak and lockdown among students and workers of a Spanish university. Psychiatry Res..

[8] Liang L., Ren H., Cao R., Hu Y., Qin Z., Li C., Mei Z. (2020). The Effect of COVID-19 on Youth Mental Health. Psychiatr. Q..

[9] Xie X., Xue Q., Zhou Y., Zhu K., Liu Q., Zhang J., Song R. (2020). Mental health status among children in home confinement during the coronavirus disease 2019 outbreak in Hubei Province, China. JAMA Pediatr..

[10] Holmes E.A., O’Connor R.C., Perry V.H., Tracey I., Wessely S., Arseneault L., Ballard C., Christensen H., Silver R.C., Everall I. (2020). Multidisciplinary research priorities for the COVID-19 pandemic: A call for action for mental health science. Lancet Psychiatry.

[11] Jones S.E., Ethier K.A., Hertz M., DeGue S., Le V.D., Thornton J., Lim C., Dittus P.J., Geda S. (2022). Mental Health, Suicidality, and Connectedness among High School Students during the COVID-19 Pandemic—Adolescent Behaviors and Experiences Survey, United States, January–June 2021. MMWR Suppl..

[12] Molina R., Sepúlveda R., Carmona R., Molina T., Mac-Ginty S. (2016). Health-related quality of life in first-year college students. Rev. Chil. Public Health.

[13] Riiser K., Helseth S., Haraldstad K., Torbjørnsen A., Richardsen K.R. (2020). Adolescents’ health literacy, health-protective measures, and health-related quality of life during the COVID-19 pandemic. PLoS ONE.

[14] Nguyen H.C., Nguyen M.H., Do B.N., Tran C.Q., Nguyen T.T.P., Pham K.M., Pham L.V., Tran K.V., Duong T.T., Tran T.V. (2020). People with Suspected COVID-19 Symptoms Were More Likely Depressed and Had Lower Health-Related Quality of Life: The Potential Benefit of Health Literacy. J. Clin. Med..

[15] Pulvirenti F., Cinetto F., Milito C., Bonanni L., Pesce A.M., Leodori G., Garzi G., Miglionico M., Tabolli S., Quinti I. (2020). Health-Related Quality of Life in Common Variable Immunodeficiency Italian Patients Switched to Remote Assistance during the COVID-19 Pandemic. J. Allergy Clin. Immunol. Pract..

[16] Nobari H., Fashi M., Eskandari A., Villafania S., Murillo Á., Pérez J. (2021). Effect of COVID-19 on Health-Related Quality of Life in Adolescents and Children: A Systematic Review. Int. J. Environ. Res. Public Health.

[17] Vazquez D.A., Caetano S.C., Schlegel R., Lourenço E., Nemi A., Slemian A., Sanchez Z.M. (2022). Schoolless life and mental health of public-school students in the COVID-19 pandemic. Saúde Debate.

[18] Pharr J.R., Coughenour C., Gakh M., Bungum T., Jalene S., Whitehead M., Sharma M. (2022). Predictors of Depression among College Students in the Early Stages of the COVID-19 Pandemic. Glob. J. Health Sci..

[19] Puteikis K., Mameniškytė A., Mameniškienė R. (2022). Sleep Quality, Mental Health and Learning among High School Students after Reopening Schools during the COVID-19 Pandemic: Results of a Cross-Sectional Online Survey. Int. J. Environ. Res. Public Health.

[20] Hidalgo C.A., Rajmil L., Montaño R. (2021). Cross-cultural adaptation of the KIDSCREEN questionnaire to measure health-related quality of life in a Mexican population aged 8 to 18 years. Sci. Health Collect..

[21] Pinto D., Villagra D.A., Moya J.M., del Campo J., Pires R. (2014). Health-related quality of life in Latin American adolescents. Rev. Panam. Public Health.

[22] Vélez R., López S., Rajmil L. (2009). Gender and perceived health in childhood and adolescence in Spain. Sanit. Gaz..

[23] Kovacs M. (1992). Children Depression Inventory CDI (Manual).

[24] Castañeda A.D., Cardona D., Cardona J.A. (2017). Quality of life and depressive symptomatology in vulnerable adolescent women. Behav. Psychol..

[25] Guessoum S.B., Lachal L., Radjack R., Carretier E., Minassian S., Benoit L., Moro M.R. (2020). Adolescent psychiatric disorders during the COVID-19 pandemic and lockdown. Psychiatry Res..

[26] Paricio R., Pando M.F. (2020). Child and adolescent mental health and the Covid-19 pandemic in Spain: Issues and challenges. J. Child Adolesc. Psychiatry.

[27] Duan L., Shao X., Wang Y., Huang Y., Miao J., Yang X., Zhu G. (2020). An investigation of mental health status of children and adolescents in China during the outbreak of COVID-19. J. Affect. Disord..

[28] Hernández J.P., Juanico B., Juanico G., Salgado M.A., Zaragoza I. (2019). Depression and associated factors in children and adolescents from 7 to 14 years of age. Fam. Care.

[29] González C., Solís S., Juárez F., Jiménez A., Hernández G., Fernández H., Medina M.A. (2019). Depressive disorder and psychosocial indicators in high school and college students from Mexico City: Data from two censuses. Ment. Health.

[30] Berra S., Tebé C., Esandi M.E., Carignano C. (2013). Reliability and validity of the KIDSCREEN-52 questionnaire to measure health-related quality of life for the Argentine population aged 8 to 18 years. Argent. Arch. Pediatr..

[31] Ping W., Zheng J., Niu X., Guo C., Zhang J., Yang H., Shi Y. (2020). Evaluation of health-related quality of life using EQ-5D in China during the COVID-19 pandemic. PLoS ONE.

[32] Azzam N., Aljebreen A., Almuhareb A., Almadi M. (2020). Disability and quality of life before and during the COVID-19 outbreak: A cross-sectional study in inflammatory bowel disease patients. Saudi J. Gastroenterol..

